# Outcomes of Estrogen Receptor Negative and Progesterone Receptor Positive Breast Cancer

**DOI:** 10.1371/journal.pone.0132449

**Published:** 2015-07-10

**Authors:** Melissa Chan, Martin C. Chang, Rosa González, Belinda Lategan, Elvira del Barco, Francisco Vera-Badillo, Paula Quesada, Robyn Goldstein, Ignacio Cruz, Alberto Ocana, Juan J. Cruz, Eitan Amir

**Affiliations:** 1 Division of Medical Oncology and Hematology, Princess Margaret Cancer Centre, Toronto, ON, Canada; 2 Department of Medicine, University of Toronto, Toronto, ON, Canada; 3 Department of Pathology and Laboratory Medicine, Mount Sinai Hospital, Toronto, ON, Canada; 4 Department of Laboratory Medicine and Pathobiology, University of Toronto, Toronto, ON, Canada; 5 Escuela de Enfermeria, Universidad de Salamanca, Salamanca, Spain; 6 Medical Oncology Department, Salamanca University Hospital, Salamanca, Spain; 7 Servicio de Cardiología, Complejo Hospitalario de Salamanca, Salamanca, Spain; 8 Medical Oncology Department and Translational Research Unit, Albacete University Hospital, Albacete, Spain; Wayne State University School of Medicine, UNITED STATES

## Abstract

**Purpose:**

To describe the clinical features and outcomes of estrogen receptor negative (ER-) and progesterone receptor positive (PgR+) breast cancer.

**Methods:**

We retrospectively reviewed a well-characterized database of sequential patients diagnosed with early stage invasive breast carcinoma. Outcomes of interest were time to relapse (TTR) and overall survival (OS). Multivariable Cox proportional hazards analysis was conducted to assess the association of ER-/PgR+ with TTR and OS in comparison to ER+ and to ER- and PgR negative (ER-/PgR-) tumors irrespective of HER2 status. ER and PgR expression was conservatively defined as 10% or greater staining of cancer cells.

**Results:**

815 patients were followed for a median of 40.5 months; 56 patients (7%) had ER-/PgR+, 624 (77%) had ER+ and 136 (17%) had ER-/PgR- phenotypes. Compared with ER+ tumors, ER-/PgR+ tumors were associated with younger age (50 versus 59 years, p=0.03), high grade (50% versus 24%, p<0.001) and more frequent HER2 overexpression/amplification (43% versus 14%, p<0.001). TTR for ER-/PgR+ was intermediate between ER+ and ER-/PgR- tumors, but was not significantly different from ER+ tumors. Recurrences in the ER-/PgR+ and ER-/PgR- groups occurred early in follow-up while in ER+ tumors recurrences continued to occur over the duration of follow-up. OS of ER-/PgR+ was similar to ER+ tumors and better than that of ER-/PgR- tumors.

**Conclusions:**

The ER-/PgR+ phenotype is associated with higher grade with HER2 overexpression/amplification and occurs more commonly in younger women. Risk of relapse and death more closely resembles ER+ than ER-/PgR- tumors suggesting this phenotype represents a group of more aggressive hormone receptor positive tumors.

## Introduction

Hormone receptor (HR) expression in invasive breast carcinoma has both prognostic and predictive significance; the use of endocrine therapy in HR-positive breast carcinomas has been shown to reduce the rates of recurrence and mortality [1]. Estrogen receptor (ER) and progesterone receptor (PgR) status are strongly associated with each other, with PgR expression reliant on ER signalling. Consequently, the most common breast cancer phenotype expresses both ER and PgR, accounting for over 50% of all breast cancers [1]. Invasive breast cancers expressing one, but not both HRs are less common, comprising approximately 15–20% of all breast cancers. This group is comprised predominantly of tumors expressing ER, but not PgR (ER+/PgR-), a phenotype associated with greater growth factor signalling and reduced endocrine sensitivity [2–4]. Tumors expressing PgR, but not ER (ER-/PgR+) are uncommon, comprising 2–8% of breast cancers [5–7], with less known about their characteristics and responsiveness to therapy.

The clinical and biological significance of ER-/PgR+ breast cancers has been debated. Some authors hypothesize that this phenotype is a technical artifact. Several factors may alter the HR status of a breast cancer, resulting in a false-negative ER and/or false-positive PgR assay [8, 9]. These factors include improper tissue fixation, antibody selection for ER testing, different thresholds for reporting immunostaining, or less sensitive immunohistochemistry techniques [8–11]. It has been recommended by the American Society of Clinical Oncology/College of American Pathologists that ER-/PgR+ tumors should have testing repeated to rule out a false negative ER result [12].

Despite concerns about the reproducibility of the ER-/PgR+ subtype, several studies have found that this particular phenotype may represent a distinct entity. ER-/PgR+ breast tumors have different tumor and patient characteristics, when compared to tumors expressing both ER and PgR (ER+/PgR+) as well as those without expression of either HR (ER-/PgR-). Specifically, data support that ER-/PgR+ tumors occur more commonly in younger, premenopausal women and have been associated with more aggressive behaviour than ER+/PgR+ disease [5, 13]. Of interest, in the Early Breast Cancer Trialists' Collaborative Group (EBCTCG) analysis, there was a non-significant association with reduced recurrences with adjuvant tamoxifen in the ER-/PgR+ phenotype despite this group being considered ER-negative [1].

In this study, we aimed to assess the clinicopathologic features, natural history, and outcomes of ER-/PgR+ breast carcinomas and compared these to ER+ and ER-/PgR- tumors, using a retrospectively collected individual patient dataset. We hypothesized that ER-/PgR+ tumors are a true biologic entity, with a prognosis in between that seen in ER+ and ER-/PgR- tumors.

## Materials and Methods

### Study population

The study comprised sequential patients with early stage (stage I-III) breast cancer diagnosed at the University Hospital of Salamanca from 01 February 1997 to 30 December 2007. Patients with *in-situ* disease and those presenting with *de novo* metastatic disease were excluded from the study. Patients were treated per institutional guidelines and followed up once every 3–4 months for the first 3 years, every 6 months until the fifth year, and every 12 months thereafter. The University Hospital of Salamanca Institutional Review Board on Human Research approved the study. Participants were not required to provide informed consent as only de-identified information was collected.

### Data collection

Patients were identified through electronic searches of the hospital diagnostic and treatment databases. Data were retrospectively extracted from the charts of patients diagnosed with breast cancer. After confirmation of eligibility, the following data were extracted from the medical charts if available: age, breast cancer risk factors, menopausal status, tumor histological subtype, hormone receptor and HER2 status, tumor size, tumor grade, presence of positive lymph nodes, type of surgery (breast conservation or mastectomy) and the type of adjuvant treatment including chemotherapy, radiation and endocrine therapy. Follow up information was collected with specific attention to dates of last follow-up, disease recurrence or death from breast cancer or any other cause. Information was extracted by data managers of the medical oncology service under the supervision of a medical oncologist (JJC) using predesigned datasheets.

### Assessment of receptor status

All patients included in this study had the ER, PgR and HER2 status of their tumor determined in a single laboratory. ER and PgR status was assessed by semi-quantitative immunohistochemistry using the Dako ER/PR pharmDx Kit (Agilent España, Madrid, Spain). Receptor status was reported either prospectively or retrospectively using the Allred score [14]. HER2 was assessed using immunohistochemistry or fluorescence in-situ hybridization (FISH). Methods for determination of HER2 differed during the duration of the study as they were based on methods used in randomized trials of trastuzumab. Between 1997 and 2005, immunohistochemical scores of 0 or 1+ were considered negative and scores of 2+ and 3+ were considered positive [15]. Subsequently, tumors scored as 2+ were reflex tested using FISH and positive result was defined as either a HER2 signal of 6 or greater or a HER2 to CEP17 ratio of 2.0 or more [16, 17]. As the study cohort pre-dated the first American Society for Clinical Oncology/College of American Pathologists (ASCO/CAP) guidelines for the assessment of ER and PgR [12] or HER [18, 19], local guidelines were used for ER, PgR and HER2 reporting. HR expression was defined as presence of ≥10% staining of any intensity for either ER or PgR. Patients with “low-positive” staining for ER or PgR as defined by the current ASCO/CAP (1–9% staining of any intensity) were considered to have negative expression in line with treatment guidelines in place at the time of the cohort.

### Outcomes of interest

The outcomes of interest were time to relapse (TTR) and overall survival (OS) based on grouping determined by receptor status as follows: estrogen receptor (ER)-positive and any progesterone receptor (PgR) expression (ER-positive/PgR-any), ER-negative and PgR-positive, and ER and PgR negative. Groups were not assessed separately by HER2 status as this analysis aimed to focus on hormone receptor signalling and further categorization by HER2 status would have led to small subgroup leading to uncertainty in effect sizes. TTR was defined as the time from definitive surgery to the first occurrence of recurrent disease at any site. Patients dying without recurrence were censored at the time of last follow-up. OS was defined as the time from definitive surgery to death from any cause.

### Statistical analysis

Data were initially evaluated descriptively as means, medians and ranges. Baseline demographic and tumor characteristics were compared using the Chi-squared and Mann-Whitney U tests. Association of individual variables with TTR and OS was assessed using the Cox proportional hazard model. All variables with a P-value <0.1 in univariable analysis were retained in the multivariable model. The change of hazard rate over time was evaluated as the probability density of relapse over time. Interpolation was conducted using a locally weighted scatterplot smoothing (LOWESS) function. A sensitivity analysis was conducted to exclude patients with HER2-positive disease. All analyses were conducted using SPSS version 21.0 (IBM Corp, Armonk, NY). Statistical significance was defined as p<0.05.

## Results

### Patient characteristics

A total of 816 patients were followed for a median duration of 40.5 months (range 0.6–154 months). Of these, 56 patients (6.9%) had ER-/PgR+ breast cancers, while 624 (76.6%) had ER+ tumors irrespective of PgR expression (ER+/PgR-any) and 136 (16.7%) had ER-/PgR- tumors. When using the current ASCO/CAP criteria for hormone receptor expression [12], 5 of the 56 women classified as ER-/PgR+ (8.9%) were re-classified as ER+/PgR+ while among the 136 women categorized as ER-/PgR-, 12 (8.8%) were re-categorized as ER+/PgR+ and 1 (0.7%) as ER-/PgR+. A total of 172 patients (21.0%) were categorized as being HER2-positive. Of these, 26 patients (15.1%) received adjuvant trastuzumab. Patient and tumor characteristics based on receptor status are shown in Table 1.

**Table 1:** Characteristics of included patients by receptor group.

Variable	ER+/PgR any (N = 624)	ER-/PgR+ (N = 56)	ER-/PgR- (N = 136)
Age at diagnosis (median)	59	50	59
Tumor size (median, cm)	2.0	1.85	2.5
Nodal metastases	265 (42.5%)	31 (55.4%)	63 (46.3%)
High grade	152 (24.4%)	28 (50.0%)	103 (75.7%)
HER2 positive	87 (13.9%)	24 (42.9%)	58 (42.6%)
Hormone Therapy (any)	610 (97.8%)	50 (89.3%)	23 (16.9%)
Tamoxifen	349 (55.9%)	32 (57.1%)	14 (10.3%)
AI	261 (41.8%)	18 (32.1%)	9 (6.6%)
None	9 (1.4%)	5 (8.9%)	113 (83.1%)
Unknown	4 (0.6%)	1 (1.8%)	0 (0%)
Chemotherapy (any)	436 (69.9%)	49 (87.5%)	120 (88.2%)
Anthracycline-based	190 (30.4%)	20 (35.7%)	71 (52.2%)
Anthracycline and Taxane	150 (24.0%)	21 (37.5%)	33 (24.3%)
Taxane-based	2 (0.3%)	1 (1.8%)	0 (0%)
CMF	94 (15.1%)	7 (12.5%)	16 (11.8%)
None	179 (28.7%)	7 (12.5%)	15 (11.0%)
Unknown	9 (1.4%)	0 (0%)	1 (0.7%)
Trastuzumab	12 (13.8%)*	7 (29.2%)*	7 (12.1%)*
Locoregional radiation	455 (72.9%)	40 (71.4%)	98 (72.1%)
Recurrence (any)	84 (13.5%)	11 (19.6%)	47 (34.6%)
Local	4 (0.6%)	0 (0%)	6 (4.4%)
Regional	8 (1.3%)	1 (1.8%)	5 (3.7%)
Distant	62 (9.9%)	10 (17.9%)	36 (26.5%)

* Percentage refers to patients with HER2-positive disease.

Compared with ER+ patients, those with ER-/PgR+ tumors were younger at the time of diagnosis (median age 50 versus 59 years, p = 0.03), were more likely to be high grade (50.0% vs 24.4%, p<0.001) and more likely to have HER2-overexpressing/amplified tumors (42.9% vs. 13.9%, p<0.001). There was a non-significant association with lymph node metastases (55.4% vs. 42.5%, p = 0.09). There were no differences in median tumor size (1.85cm vs. 2.0cm, p = 0.77). Fewer patients with ER-/PgR+ disease received adjuvant endocrine therapy (89.3% vs. 97.8%, p<0.001); however, more received adjuvant chemotherapy (87.5% vs. 69.9%, p = 0.008).

Compared with ER-/PgR- patients, those with ER-/PgR+ tumours were non-significantly younger at the time of diagnosis (median age 50 versus 59 years, p = 0.28), were less likely to be high grade (50.0% vs 75.7%, p = 0.001) and had smaller median tumor sizes (1.85cm vs. 2.5cm, p = 0.01). There were no differences in the proportion with HER2-overexpressing/amplified tumors (42.9% vs. 42.6%, p = 0.95), or with nodal metastases (55.4% vs. 46.3%, p = 0.36). As expected, more patients with ER-/PgR+ disease received adjuvant endocrine therapy (89.3% vs. 16.9%, p<0.001). Some patients in the ER-/PgR- group received endocrine therapy coded as prophylactic. Similar numbers received adjuvant chemotherapy (87.5% vs. 88.2%, p = 0.79).

### Time to relapse

**Fig 1:**
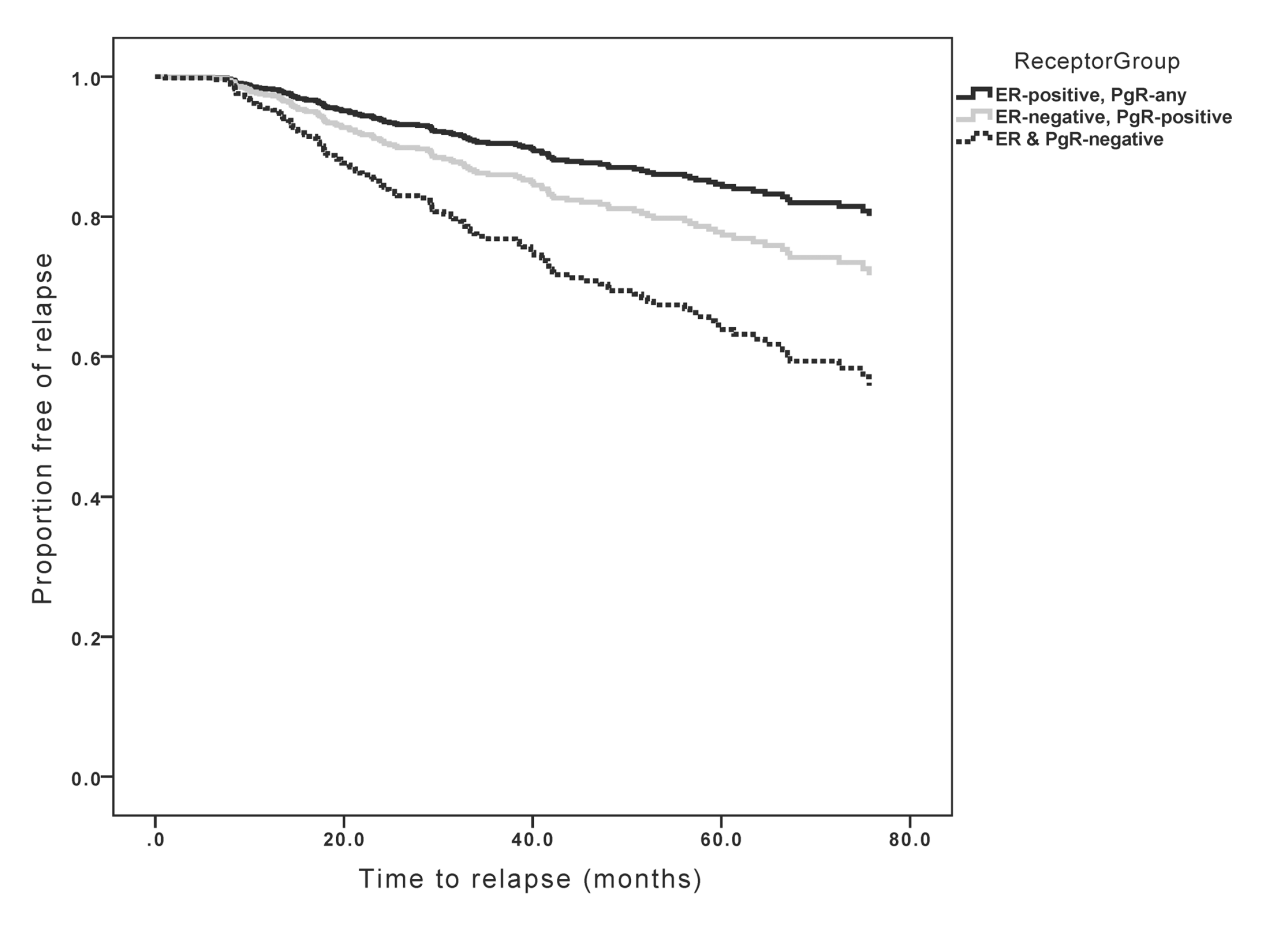
Kaplan-Meier curves for time to relapse based on receptor group.

Univariable analysis showed that tumor size, nodal metastases, grade, HR status, HER2 over-expression/amplification and receipt of adjuvant chemotherapy were associated with worse TTR. In contrast, age and receipt of adjuvant endocrine therapy were negatively associated with improved TTR (see Table 2). Sensitivity analysis excluding patients with HER2-positive disease showed generally similar results except for receipt of chemotherapy which showed no association with relapse in contrast to an association with adverse outcomes in patients unselected for HER2 status (Table 3). In multivariable analysis, tumor size, nodal metastases, grade and HR status remained statistically significant. TTR was not significantly different between ER+ and ER-/PgR+ tumors. However, patients with ER-/PgR- tumors had significantly worse TTR (Table 2 and Fig 1). Sensitivity analysis excluding patients with HER2-positive disease showed generally similar results (Table 3). Distant recurrence was overwhelmingly the first site of relapse in our cohort comprising 76.1% of all recurrences. There was no obvious difference in the sites of first recurrence (local vs. regional vs. distant) between the three receptor groups (Mann Whitney U p = 0.46).

**Table 2:** Univariable and multivariable analyses for time to relapse.

Variable	HR	95% CI	p
**Univariable Analysis**			
Age at diagnosis	0.98	0.97–0.99	<0.001
Tumour size	1.18	1.10–1.27	<0.001
Nodal metastases	2.55	1.81–3.58	<0.001
Grade	1.91	1.46–2.50	<0.001
HER2 positive	1.76	1.25–2.49	0.001
Receptor Group			
ER-positive, PgR-any	Ref		
ER-negative, PgR-positive	1.83	0.98–3.43	0.059
ER & PgR-negative	2.76	1.94–3.93	<0.001
Locoregional radiation	1.08	0.75–1.57	0.68
Chemotherapy	1.98	1.21–3.24	0.007
Hormone therapy	0.45	0.31–0.64	<0.001
**Multivariable Analysis**			
Age at diagnosis	0.97	0.96–0.98	<0.001
Tumour size	1.13	1.04–1.23	0.006
Nodal metastases	2.22	1.49–3.33	<0.001
Grade	1.36	1.00–1.85	0.048
HER2 positive	1.06	0.70–1.60	0.80
Receptor Group			
ER-positive, PgR-any	Ref		
ER-negative, PgR-positive	1.57	0.79–3.12	0.20
ER & PgR-negative	3.64	1.69–7.82	0.001
Locoregional radiation	-	-	-
Chemotherapy	0.70	0.34–1.44	0.33
Hormone therapy	1.47	0.70–3.08	0.31

**Table 3:** Univariable and multivariable analyses for time to relapse in sensitivity analysis excluding patients with HER2-positive disease.

Variable	HR	95% CI	p
**Univariable Analysis**			
Age at diagnosis	0.97	0.96–0.98	<0.001
Tumour size	1.25	1.14–1.38	<0.001
Nodal metastases	2.50	1.66–3.77	<0.001
Grade	2.11	1.52–2.93	<0.001
Receptor Group			
ER-positive, PgR-any	Ref		
ER-negative, PgR-positive	2.11	0.92–4.88	0.08
ER & PgR-negative	3.05	1.93–4.83	<0.001
Locoregional radiation	0.95	0.61–1.48	0.81
Chemotherapy	0.93	0.80–1.07	0.31
Hormone therapy	0.48	0.34–0.66	<0.001
**Multivariable Analysis**			
Age at diagnosis	0.98	0.96–0.99	0.003
Tumour size	1.26	1.10–1.44	0.001
Nodal metastases	1.81	1.12–2.92	0.02
Grade	1.450	1.04–2.17	0.03
Receptor Group			
ER-positive, PgR-any	Ref		
ER-negative, PgR-positive	1.86	0.77–4.48	0.17
ER & PgR-negative	2.07	0.91–4.68	0.08
Locoregional radiation	-	-	-
Chemotherapy	-	-	-
Hormone therapy	0.87	0.50–1.52	0.62

**Fig 2:**
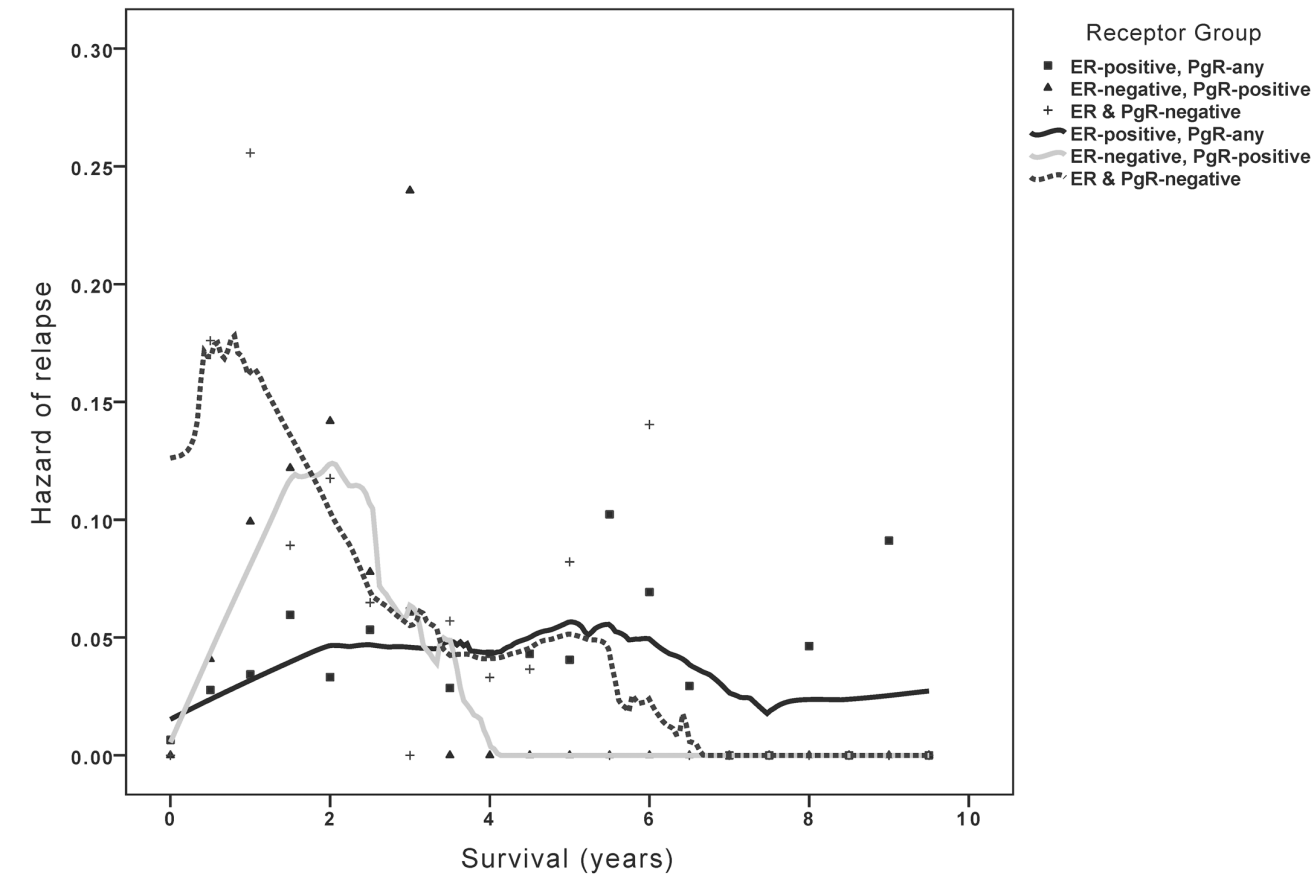
Hazard density plot depicting timing of relapse based on receptor status.

Analysis of timing of relapse showed that ER+ tumors had generally low annual hazards for relapse, but that these continued throughout the duration of follow-up. In contrast, both ER-/PgR+ and ER-/PgR- patients had higher rates of relapse, but these predominantly occurred early in follow-up with no observed relapses beyond 6 years of follow-up in either group (Fig 2).

### Overall survival

**Fig 3:**
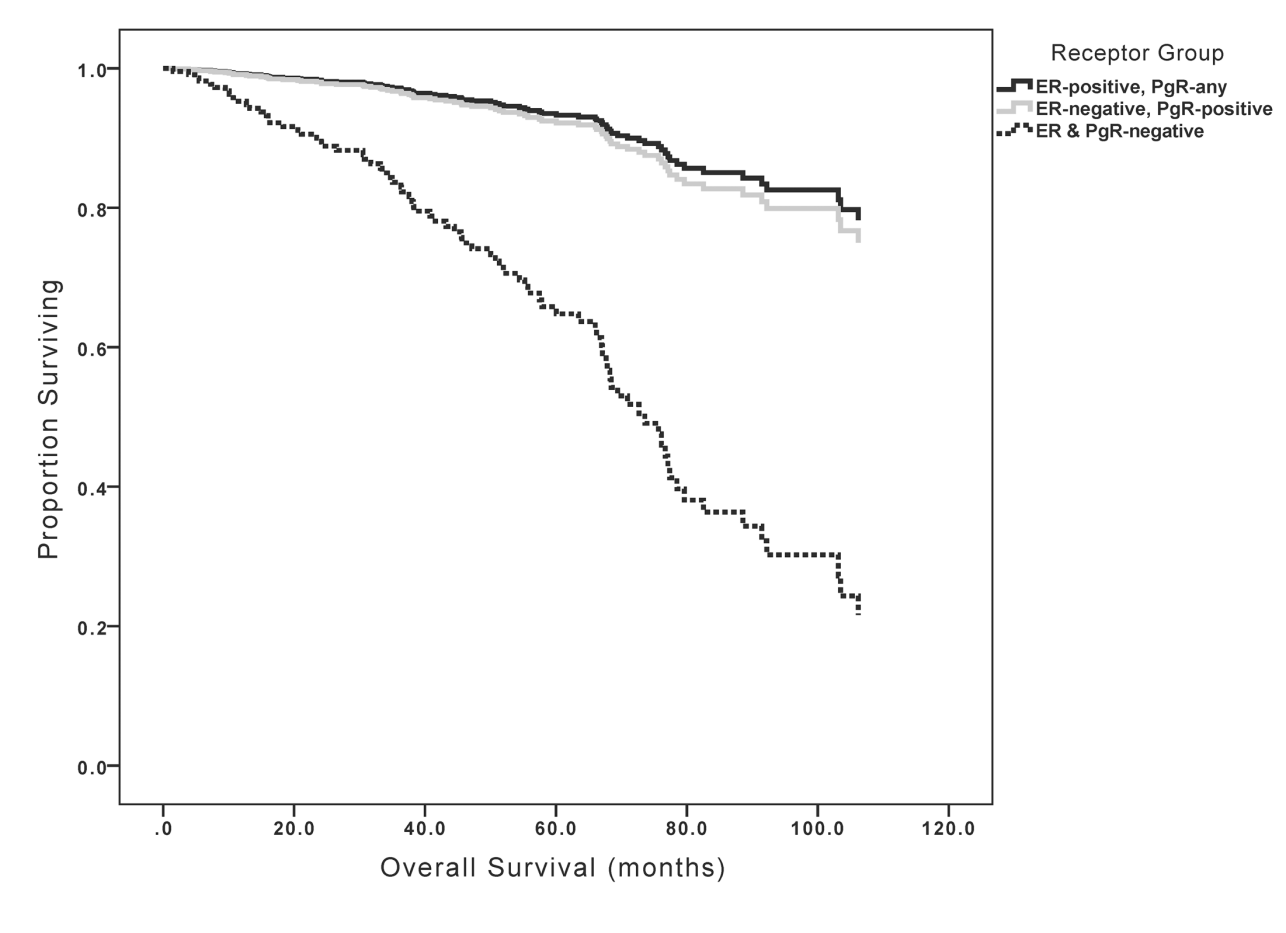
Kaplan-Meier curves for overall survival based on receptor group.

Univariable analysis showed that tumor size, nodal metastases, grade, HR status and HER2 over-expression/amplification were positively associated with worse OS. In contrast, receipt of adjuvant endocrine therapy was associated with improved OS (see Table 4). Sensitivity analysis excluding patients with HER2-positive disease showed generally similar results except for receipt of chemotherapy, which showed a significant association with improved survival in contrast to no such association in patients unselected for HER2 status (Table 5). In multivariable analysis, nodal metastases and HR status remained statistically significant. Patients with ER+ tumors and those with ER-/PgR+ tumors had similar OS, but those with ER-/PgR- tumors had significantly worse OS (Table 4 and Fig 3). Sensitivity analysis excluding patients with HER2-positive disease showed generally similar results including receipt of chemotherapy which retained its significant association with improved survival (Table 5).

**Table 4:** Univariable and multivariable analyses for overall survival.

Variable	HR	95% CI	p
**Univariable analysis**			
Age at diagnosis	1.01	1.00–1.02	0.20
Tumour size	1.23	1.14–1.34	<0.001
Nodal metastases	2.02	1.33–3.07	0.001
Grade	2.27	1.57–3.29	<0.001
HER2 positive	1.67	1.10–2.54	0.02
Receptor Group			
ER-positive, PgR-any	Ref		
ER-negative, PgR-positive	1.10	0.40–3.06	0.85
ER & PgR-negative	3.67	2.43–5.53	<0.001
Locoregional radiation	1.10	0.70–1.74	0.67
Chemotherapy	0.72	0.45–1.14	0.16
Hormone therapy	0.42	0.27–0.64	<0.001
**Multivariable analysis**			
Age at diagnosis	-	-	-
Tumour size	1.10	0.99–1.23	0.10
Nodal metastases	1.72	1.06–2.79	0.03
Grade	1.38	0.90–2.11	0.14
HER2 positive	0.82	0.49–1.36	0.43
Receptor Group			
ER-positive, PgR-any	Ref		
ER-negative, PgR-positive	1.21	0.42–3.48	0.73
ER & PgR-negative	5.89	2.66–13.04	<0.001
Locoregional radiation	-	-	-
Chemotherapy	-	-	-
Hormone therapy	1.88	0.90–3.94	0.10

**Table 5:** Univariable and multivariable analyses for overall survival in sensitivity analysis excluding patients with HER2-positive disease.

Variable	HR	95% CI	p
**Univariable Analysis**			
Age at diagnosis	1.02	1.00–1.04	0.03
Tumour size	1.31	1.19–1.45	<0.001
Nodal metastases	1.78	1.07–2.96	0.03
Grade	2.14	1.38–3.32	0.001
Receptor Group			
ER-positive, PgR-any	Ref		
ER-negative, PgR-positive	2.30	0.82–6.46	0.11
ER & PgR-negative	4.29	2.51–7.33	<0.001
Locoregional radiation	0.96	0.55–1.66	0.87
Chemotherapy	0.76	0.62–0.94	0.01
Hormone therapy	0.60	0.39–0.94	0.02
**Multivariable Analysis**			
Age at diagnosis	1.03	1.00–1.05	0.02
Tumour size	1.26	1.05–1.52	0.02
Nodal metastases	1.58	0.82–3.02	0.17
Grade	1.22	0.73–2.04	0.44
Receptor Group			
ER-positive, PgR-any	Ref		
ER-negative, PgR-positive	4.49	1.39–14.48	0.01
ER & PgR-negative	7.35	3.04–17.77	<0.001
Locoregional radiation	-	-	-
Chemotherapy	0.72	0.55–0.94	0.02
Hormone therapy	1.27	0.70–2.32	0.44

## Discussion

HR status is an important prognostic and predictive factor in breast cancer. While most HR expressing breast cancers express the ER, a small minority express PgR but not ER, even after retesting. There remains uncertainty about whether the ER-/PgR+ phenotype is real or simply an analytic artifact. The ER-/PgR+ subgroup may represent false negative ER, which may result from failed binding of the ER by the antibody used for its detection. Such lack of binding may result from a conformational change of the ER, caused, for example, by ER mutations, which may change the receptor to the extent that antibody-antigen binding is not achieved or due to competitive antagonism of the ER by another molecule in the tumor microenvironment, preventing the antibody from binding to ER. Alternatively, this subgroup may represent false positive PgR; anti-PgR antibodies may cross-react with other antigens. To avoid these false results, analysis of ER and PgR status with two independent antibodies is desirable as the epitopes recognized by different antibodies should be distinct.

In this study, we have utilized a well-maintained, retrospectively collected dataset of sequential breast cancer patients to explore the clinicopathological characteristics and outcomes of patients with ER-/PgR+ breast cancer. In our cohort, less than 7% of patients fulfilled the ER-/PgR+ phenotype. These patients were younger than both ER+ and ER-/PgR- subgroups, and exhibited less favorable prognostic factors compared to women with ER+ disease, including higher grade, more frequent nodal metastases and HER2 over-expression/amplification.

The association between the ER-/PgR+ subgroup and younger age has been described previously [20, 21]. This association is also supported by laboratory data that show that PgR expression is more common in breast cancers diagnosed in pre-menopausal women compared to those diagnosed after menopause [22]. The differential regulation of ER may explain the occurrence of ER-/PgR+ breast tumors in younger women. The expression of ER is a complex process subject to hormonal regulation by varying estrogen levels. Higher estrogen levels in menstruating women appear to downregulate the ER protein [23]. Other proposed molecular mechanisms that may occur more commonly in younger women include the epigenetic regulation of ER expression and the activation of HER2 receptors strongly downregulating ER. Taken as a whole, these data support the ER-/PgR+ phenotype as a real phenomenon occurring in a distinct group of younger patients.

Our data also showed that despite more frequent poor prognostic factors, both TTR and OS of women with ER-/PgR+ tumors were not significantly different from those of ER+ disease. However, the timing of relapse was substantially different; women with ER-/PgR+ tumors showed a higher hazard of relapse early in follow-up with no observed relapses beyond year 6 after diagnosis. Conversely, women with ER+ tumors showed lower hazards for relapse and these hazards persisted for the duration of follow-up similar to data reported elsewhere [24, 25]. These data may explain why women with ER-negative and PgR-positive disease in the EBCTCG meta-analysis showed a trend towards initial benefit from adjuvant tamoxifen, an observation which was lost with longer follow-up. Of interest, when compared to ER-/PgR- tumors, those with ER-/PgR+ disease showed more favourable outcomes despite similar prognostic factors suggesting that the ER-/PgR+ phenotype possesses characteristics of more proliferative endocrine-sensitive tumors.

This study has limitations. First, this was a retrospective study with a relatively short median follow-up time. With HR expressing breast cancer being associated with recurrences many years after diagnosis, it is likely that a number of recurrences occurring at a later time may not have been captured. Complete follow-up of all patients was also not available. Second, we elected to use a conservative cut-off for defining HR expression. The current ASCO/CAP defines HR expression as the presence of at least 1% of invasive cancer cells staining for either ER or PgR. In this study we utilized a cut-off of 10% as the purpose was to achieve a high positive predictive value for endocrine sensitivity. The choice of this cut-off may have affected the negative predictive value for identification of endocrine sensitivity. Finally, our analysis of HER2 is limited due to the differences in the treatment of HER2 overexpressing or amplified disease in our cohort. Adjuvant trastuzumab became available in the last year of inception of our cohort and only a minority of HER2-positive patients received this agent. Sensitivity analysis excluding HER2-positive patients showed similar results. However, the contemporary validity of our data with regard to HER2-positive patients treated with adjuvant trastuzumab remains unclear.

In summary, ER-/PgR+ tumors are a rare, but defined subgroup of breast cancer occurring more frequently in younger women. The risk of relapse and death more closely resembles ER+ than ER-/PgR- tumors suggesting this phenotype represents a group of more aggressive hormone receptor positive tumors. The reasons of PgR expression in the absence of ER expression remains unclear and further research in this setting is warranted especially as it relates to younger, premenopausal women.
